# Expression of *Ralstonia solanacearum* type III secretion system is dependent on a novel type 4 pili (T4P) assembly protein (TapV) but is T4P independent

**DOI:** 10.1111/mpp.12930

**Published:** 2020-03-20

**Authors:** Yong Zhang, Liangliang Han, Lichun Zhang, Changzheng Xu, Xiaojun Shi, Yasufumi Hikichi, Kouhei Ohnishi

**Affiliations:** ^1^ College of Resources and Environment Southwest University Chongqing China; ^2^ Key Laboratory of Efficient Utilization of Soil and Fertilizer Resources Chongqing China; ^3^ Research Institute of Molecular Genetics Kochi University Kochi Japan; ^4^ College of Life science Southwest University Chongqing China; ^5^ Laboratory of Plant Pathology and Biotechnology Kochi University Kochi Japan

**Keywords:** pathogenesis, *Ralstonia solanacearum*, TapV, type 4 pili, type III secretion system

## Abstract

Type IV pili (T4P) are virulence factors in various pathogenic bacteria of animals and plants that play important roles in twitching motility, swimming motility, biofilm formation, and adhesion to host cells. Here, we genetically characterized functional roles of a putative T4P assembly protein TapV (Rsc1986 in reference strain GMI1000) and its homologue Rsp0189, which shares 58% amino acid identity with TapV, in *Ralstonia solanacearum*. Deletion of *tapV*, but not *rsp0189*, resulted in significantly impaired twitching motility, swimming motility, and adhesion to tomato roots, which are consistent as phenotypes of the *pilA* mutant (a known *R. solanacearum* T4P‐deficient mutant). However, unlike the *pilA* mutant, the *tapV* mutant produced more biofilm than the wild‐type strain. Our gene expression studies revealed that TapV, but not Rsp0189, is important for expression of a type III secretion system (T3SS, a pathogenicity determinant of *R. solanacearum*) both in vitro and in planta, but it is T4P independent. We further revealed that TapV affected the T3SS expression via the PhcA–TapV–PrhG–HrpB pathway, consistent with previous reports that PhcA positively regulates expression of *pilA* and *prhG*. Moreover, deletion of *tapV*, but not *rsp0189*, significantly impaired the ability to migrate into and colonize xylem vessels of host plants, but there was no alteration in intercellular proliferation of *R. solanacearum* in tobacco leaves, which is similar to the *pilA* mutant. The *tapV* mutant showed significantly impaired virulence in host plants. This is the first report on the impact of T4P components on the T3SS, providing novel insights into our understanding of various biological functions of T4P and the complex regulatory pathway of T3SS in *R. solanacearum*.

## Graphical abstract

A novel type 4 pili (T4P) assembly protein (TapV) was identified to contribute to full virulence of *Ralstonia solanacearum*, which affects the T3SS expression via the PhcA–TapV–PrhG–HrpB pathway but is T4P independent.

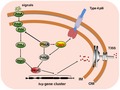

## 1 INTRODUCTION

Pili or fimbriae are hair‐like appendages found on the surface of a wide range of bacteria (Burdman *et al.*, 2011; Dunger *et al.*, 2016). There are several types of pili that differ in their mechanisms of assembly, structure, and function. Type IV pili (T4P) are the most abundant pili and the best studied thus far (Mattick, 2002; Gibiansky *et al*., 2010). T4P are proteinaceous, flexible filaments with a diameter of 5–8 nm and a length of several micrometres that are generally located at one or both poles of a cell (Strom *et al*., 1993; Fernandez and Berenguer, 2000). T4P are mainly composed of thousands of copies of a small (13–23 kDa) subunit named pilin (PilA in most cases) that are synthesized as prepilin and cleaved by the action of PilD to make the mature pilin. PilA units are assembled into pilins by the cytoplasmic membrane protein PilC to form an extracellular helical polymer via the outer membrane secretin PilQ (Craig *et al.*, 2004, 2019; Craig and Li, 2008). Several components of PilC, PilM, PilN, PilO, and PilP form the inner membrane platform that interacts with pilins and two specialized hexameric ATPases of PilB and PilT on the cytosolic face of the inner membrane (Craig *et al.*, 2004; Craig and Li, 2008; Burdman *et al.*, 2011).

To date, functions of T4P and several T4P‐dependent specific phenotypes have been well characterized in various bacteria such as *Pseudomonas*, *Neisseria*, *Escherichia*, *Vibrio*, *Xylella*, and *Xanthomonas* spp. (Gibiansky *et al*., 2010; Burdman *et al.*, 2011; Dunger *et al.*, 2016). Adherence to eukaryotic cells, an important early step during infection of host cells by many pathogenic bacteria, is one of the original functions of T4P (Henderson *et al.*, 1999; Finlay and Caparon, 2000; Burdman *et al.*, 2011). Twitching motility, the most representative T4P‐dependent phenotype, is a form of bacterial translocation over moist organic and inorganic surfaces. It is an efficient and versatile flagellar‐independent form of bacterial surface motility that is promoted by extension, attachment, and subsequent retraction of T4P in many different bacteria (Wall *et al.*, 1999; Merz *et al.*, 2000; Skerker *et al.*, 2001; Maier and Wong, 2015). A remarkable feature of twitching motility is the edge of the expanding colonies, termed the twitching zone, which is made up of small groups or individual cells with poorly defined and irregular boundaries. T4P mutants usually present a more uniform and well‐defined boundary with tightly packed cells (Bradley, 1980; Wall and Kaiser, 2010). Besides adherence to host cells and twitching motility, T4P also play important roles in surface attachment, biofilm formation, genetic material uptake, and bacteriophage infection (Kang *et al*, 2002; Gibiansky *et al*., 2010; Dunger *et al.*, 2016).

Over the past few decades, the function, structure, and regulation of T4P have been well studied in genera of *Pseudomonas*, *Neisseria*, *Escherichia*, and *Vibrio*, where they are essential virulence factors of many human pathogenic bacteria (Burdman *et al.*, 2011; Dunger *et al.*, 2016), while they are less studied in plant pathogenic bacteria except for *Xylella* and *Xanthomonas* species, belonging to the *Xanthomonadaceae* family (Hu *et al.*, 1995; Yang *et al.*, 2004; Meng *et al.*, 2005; Li *et al.*, 2007). Although pioneering work has provided several links between T4P and *Xanthomonas* physiology and virulence, the role of T4P in the pathogenicity of plant pathogenic bacteria is poorly understood (Burdman *et al.*, 2011; Dunger *et al.*, 2016). In plant pathogenic bacteria, the contribution of T4P to virulence has mainly been investigated in a few vascular pathogens, including *Ralstonia solanacearum*, where it may contribute to colonization and dispersal in xylem vessels through cell attachment, biofilm formation, and twitching motility (Burdman *et al.*, 2011; Dunger *et al.*, 2016). *R. solanacearum*, the causal agent of bacterial wilt disease in many plant species worldwide, has been employed as a model system to decipher molecular interactions between plant and pathogenic bacterium (Genin and Denny, 2012; Jiang *et al.*, 2017), although there are only a few studies to date that demonstrate the contribution of T4P to its virulence (Liu *et al.*, 2001; Kang *et al.*, 2002; Wairuri *et al.*, 2012).

As a soilborne vascular bacterium, *R. solanacearum* generally invades host plants through natural root openings or root wounds (Vasse *et al.*, 1995; Janse *et al.*, 2004). Once it has invaded xylem vessels, it proliferates extensively and produces a huge number of exopolysaccharides (EPS) to block sap flow, resulting in quick stunting and wilting (Roberts *et al.*, 1988; Denny, 1995). A syringe‐like type III secretion system (T3SS) is another essential pathogenicity determinant in *R. solanacearum*, which many pathogenic bacteria use to inject virulence factors (type III effectors, T3Es) into host cytosol to subvert host defence (Cunnac *et al.*, 2004; Angot *et al.*, 2006; Jones and Dangl, 2006). The T3SS in *R. solanacearum* is encoded by 22 genes forming an *hrp* regulon and is globally regulated by a complex network (Arlat *et al.*, 1992; Hikichi et al., 2017; Genin and Denny, 2012). In general, a master regulator HrpB of the AraC family of transcriptional regulators directly controls the T3SS and T3Es (Mukaihara *et al.*, 2010; Coll and Valls, 2013). Expression of the T3SS and *hrpB* is not activated until the bacterium comes into contact with host signals or some mimic signals, such as those in nutrient‐limited medium that mimics plant apoplastic fluids (Marenda *et al.*, 1998; Yoshimochi *et al.*, 2009; Zhang *et al.*, 2013). Expression of *hrpB* is positively regulated by HrpG and PrhG in a parallel way. These are two close paralogs of two‐component system response regulators and can respond to host signals by phosphorylation (Plener *et al.*, 2010; Zhang *et al.*, 2013). Host signals or some mimic signals are presumed to be recognized by an outer membrane PrhA and transferred to HrpG via a signalling cascade of PrhA‐PrhR/I‐PrhJ or some novel cascades (Valls *et al.*, 2006; Genin and Denny, 2012; Hikichi *et al.*, 2017; Zhang *et al.*, 2018). Besides HrpG and PrhG, the global regulation network also includes numerous well‐studied regulators such as PhcA, PrhN, PrhO, and XpsR cascades (Valls *et al.*, 2006; Genin and Denny, 2012; Hikichi *et al.*, 2017; Zhang *et al.*, 2018).

To further elucidate the global regulation of T3SS in *R. solanacearum*, we previously screened several T3SS‐regulating candidates with transposon mutagenesis, in which expression profiles of the T3SS were monitored with a *popA‐lacZYA* fusion (Zhang *et al.*, 2013). Among these was Rsc1986 (962 amino acids in the reference strain GMI1000), (https://iant.toulouse.inra.fr/bacteria/annotation/cgi/ralso.cgi), which is annotated as a putative T4P‐assembly protein TapV, sharing 30% amino acid identity to known Tap proteins. Tap proteins were originally identified in *Aeromonas* species and abbreviated as type IV Aeromonas pilus (Tap), in which T4P is encoded in part by the *tapABCD* operon (Barnett and Kirov, 1999; Boyd *et al.*, 2008). Rsp0189 (673 amino acids) was identified as a homolog of TapV, sharing 58% amino acid identity. Expression of T3SS was substantially reduced in *tapV* transposon mutants, and is thus the first report linking the T3SS and T4P components. We therefore focused on TapV and Rsp0189 to investigate their roles in T4P properties, T3SS regulation, and contribution to pathogenicity in *R. solanacearum*.

## 2 RESULTS

### 2.1 Structural features of TapV and Rsp0189

Genomes of *R. solanacearum* strains possess a unique feature of two replicons: one chromosome (3.7 Mb in GMI1000) and one megaplasmid (2.1 Mb in GMI1000) (Salanoubat *et al*., 2002). TapV (Rsc1986 in GMI1000) is located in the chromosome of GMI1000, but Rsp0189 in the megaplasmid. *R. solanacearum* strains are extremely heterogeneous, while TapV and Rsp0189 are greatly conserved, exhibiting more than 95%  amino acid identity among different *R. solanacearum* strains. TapV and Rsp0189 appear to be orphans because they do not transcriptionally link with other genes and no transposase or integrase have been identified nearby, indicating that these two genes are not integrated by simple transposition or horizontal gene transfer (https://iant.toulouse.inra.fr/bacteria/annotation/cgi/ralso.cgi). For instance, TapV is located between two genes encoding aspartate‐semialdehyde dehydrogenase (Rsc1987) and tRNA pseudouridine synthase A (Rsc1985), and clear intergenic spacers can be observed between these genes. Moreover, TapV and Rsp0189 exhibit quite high guanine and cytosine (GC) contents of 70.8% and 71.2%, respectively, which is consistent with about 69% of GC content in the genome of *R. solanacearum* GMI1000 (Salanoubat *et al*., 2002).

**FIGURE 1:**
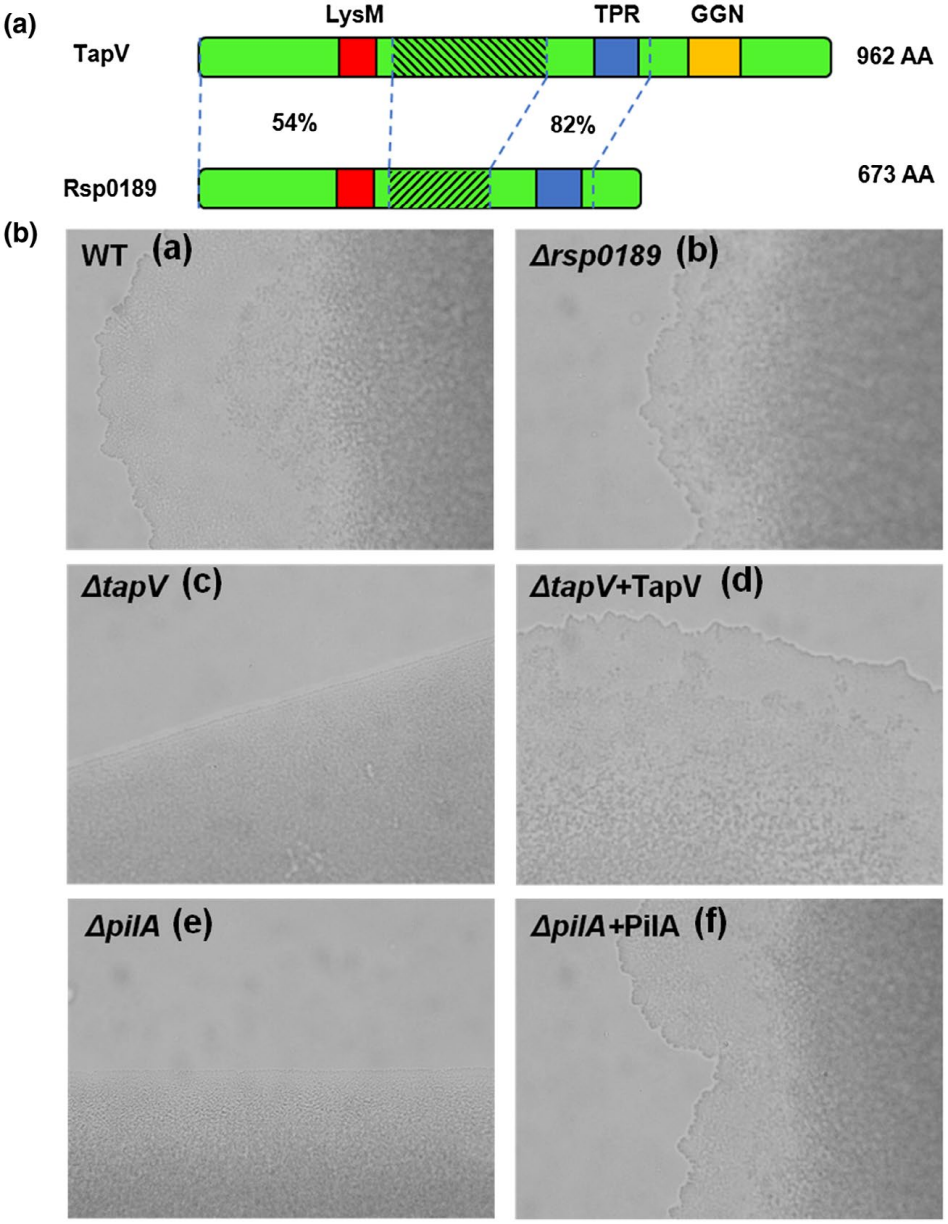
Involvement of TapV on twitching motility of *Ralstonia solanacearum*. (a) Schematic and comparison of TapV (Rsc1986 in reference strain GMI1000, 962 amino acids, AA) and its homologue Rsp0189 (673 amino acids). TapV and Rsp0189 contain a peptidoglycan (PG)‐binding domain (LysM, red) and a predicted tetratricopeptide repeat domain (TPR, grey). The figures between TapV and Rsp0189 refer to amino acid identities of two regions between TapV and Rsp0189. (b) Twitching motility assay of strains. (a) wild‐type refers to the wild‐type strain (RK5050), (b) Δ*rsp0189* refers to RQ6069 (RK5050, Δ*rsp0189*), (c) Δ*tapV* refers to RQ5703 (RK5050, Δ*tapV*), (d) Δ*tapV* + *tapV* refers to RQC0343 (RQ5703 with complementary *tapV*), (e) Δ*pilA* refers to RQ6055 (RK5050, Δ*pilA*), and (f) Δ*pilA* + *pilA* refers to RQC0347 (RQ6055 with complementary *pilA*). A bacterial suspension of 10 µl at an OD_600_ of 0.1 was dropped onto the surface of 1% broth agar on sterile slides and kept at 28 °C for 24 hr with coverslips. Twitching zones were observed with a light microscope (Olympus CX21) equipped with a 40× objective. Each assay was carried out with three biological replicates including four replications per trial. Typical twitching zones could be observed on all samples from the wild‐type strain and complementary strains, but not on any sample from the *tapV* or *pilA* mutants. The empty vector pUC18mini‐Tn*7*T‐Gm was subjected to system control, which did not affect formation of twitching zones in either of the strains (data not shown), and a representative result is presented

Blast search analysis at National Center for Biotechnology Information suggests that *R. solanacearum* TapV is a member of the FimV super family that contains a putative peptidoglycan (PG)‐binding LysM domain (residues 214–259), a putative tetratricopeptide repeat (TPR) domain (residues 602–669), and a putative gametogenetin (GGN) domain (residues 745–823) (Figure 1a). The LysM domain has been well characterized to be responsible for PG‐binding, which enables strong association with T4P components on the outer membrane (Bateman and Bycroft, 2000; Wehbi *et al.*, 2011; Sieweringa *et al*., 2014). The TPR domain is typically involved in protein–protein interactions (Dandrea and Regan, 2003; Robinson *et al*., 2005), and the GGN domain has been found to be associated with the intracellular membrane and is involved in vesicular trafficking (Strong and Schimenti, 2010). FimV has been validated to be important for T4P assembly and T4P‐dependent twitching motility in *Pseudomonas aeruginosa* (Bateman and Bycroft, 2000; Semmler *et al.*, 2000). TapV is thus assumed to be one of T4P assembly proteins that may span the membrane and interact with components of T4P. Rsp0189 (673 amino acids) has been identified as a homolog to TapV, sharing 54% of identity at N‐termini of about 300 amino acids containing the typical LysM domain, and 82% of identity in the central region of 150 amino acids containing the TPR domain (Figure 1a). It is worthwhile noting that LysM domains of TapV and Rsp0189 share 85% amino acid identity.

### 2.2 TapV but not Rsp0189 is essential for T4p‐dependent twitching motility in *R. solanacearum*

To determine whether TapV and Rsp0189 are involved in functional T4P of *R. solanacearum*, we first evaluated their requirement for twitching motility, one of the most representative T4P‐dependent features in many bacteria. Colonies of the wild‐type strain (RK5050) growing on the surface of broth agar plates exhibited typical twitching zones that are made up of small groups or individual cells with a poorly defined and irregular boundary, while colonies of the *tapV* mutant (RQ5703) had a more uniform and well‐defined boundary with tightly packed cells, which was similar to phenotypes of the *pilA* mutant (RQ6055), a well‐known T4P‐deficient mutant in many bacteria (Figure 1b). It was noteworthy that these typical twitching zones could be observed on all colonies from the wild‐type strain, but not on any colonies from *tapV* or *pilA* mutants. Different from those of the *tapV* and *pilA* mutants, colonies of the *rsp0189* mutant (RQ6069) exhibited similar twitching zones to those in the wild‐type strain (Figure 1b). Expression of complemented *tapV* or *pilA* with their native promoters in the respective mutants could fully restore the twitching motility phenotype to that of the wild‐type strain (Figure 1b). As the control, the empty vector pUC18mini‐Tn*7*T‐Gm was integrated into chromosomes of RK5050 and the *tapV*, *pilA*, and *rsp0189* mutants, which did not affect formation of twitching zones in either of the strains (data not shown). All these results confirm that TapV, but not Rsp0189, is essential for functional T4P in *R. solanacearum*.

### 2.3 TapV is important for T4p‐dependent swimming motility and root adherence, but negatively affects biofilm information in *R. solanacearum*

**FIGURE 2:**
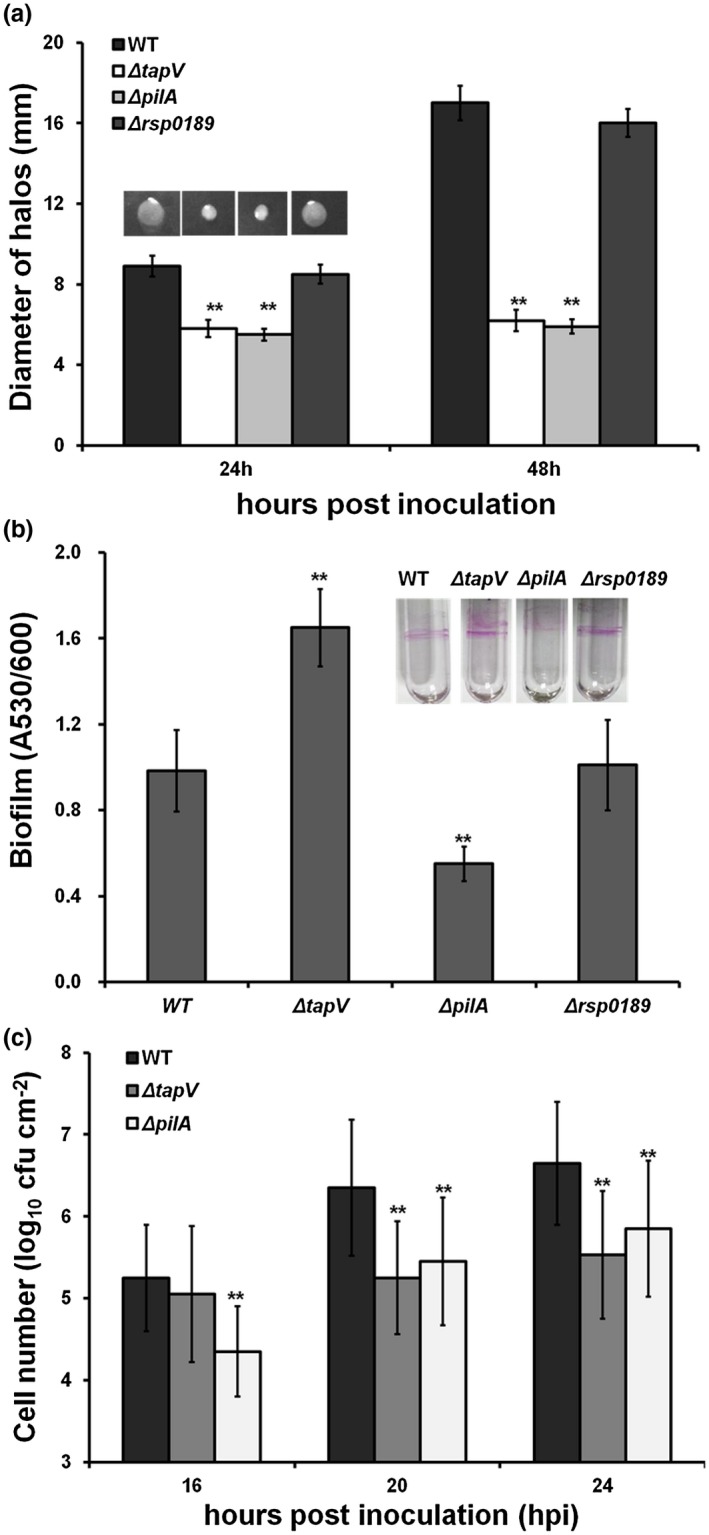
Involvement of TapV on typical T4P‐dependent properties of (a) swimming motility, (b) biofilm formation, and (c) attachment to tomato roots. Wild‐type  (WT), RK5050 (OE1‐1, *popA‐lacZYA*); Δ*tapV*, RQ5703 (RK5050, Δ*tapV*); Δ*pilA*, RQ6055 (RK5050, Δ*pilA*); Δ*rsp0189*, RQ6069 (RK5050, Δ*rsp0189*). The swimming motility assay was performed on semisolid media (0.3% agar plates) and the diameters of swimming halos on semisolid media were measured at 24 and 48 hr, respectively. The biofilm assay was carried out on polystyrene microtitre plates using the crystal violet staining method. Briefly, 20 µl of bacterial suspension at OD_600_ of 0.1 was inoculated onto 180 µl of fresh rich medium and kept at 28 °C for 24 hr without shaking. After staining with 0.1% crystal violet, biofilm formation was quantified with absorbance at 530 nm (A_530_) and normalized with OD_600_. Bacterial attachment was carried out on tomato roots. Briefly, sterile‐culture tomato roots were immersed in 10 ml of bacterial suspension at a density of 10^7^ cfu/ml and kept at 25 °C without agitation. Roots were harvested at 16, 20, and 24 hr post‐inoculation and gently dipped into water twice to remove unattached bacteria. Cell population was quantified with dilution plating and presented in log_10_ cfu/cm^2^. Each assay was carried out with at least four biological replicates, including four replications per trial*.* Mean values of all experiments were averaged with *SD*, and the statistical significance between the WT strain RK5050 and mutants (*tapV* mutant RQ5703, *pilA* mutant RQ6055 or *rsp0189* mutant RQ6069) was assessed using a post hoc Dunnett test following analysis of variance

Adherence to various surfaces, swimming motility, and biofilm formation are also typical T4P‐dependent properties in many bacteria. We assessed whether TapV is required for these T4P‐dependent properties in *R. solanacearum*. The swimming motility assay was carried out on semisolid agar plates. Swimming halos produced by the *tapV* and *pilA* mutants were significantly smaller than those produced by the wild‐type strain, while the *rsp0189* mutant produced similar swimming halos to the wild‐type strain (Figure 2a). The biofilm formation assay was carried out in polystyrene microtitre plates (96‐well plates). The *tapV* mutant formed more biofilm than the wild‐type strain, while the *rsp0189* mutant exhibited similar ability on biofilm formation to the wild‐type strain (Figure 2b), which was different from the *pilA* mutant (a known T4P‐deficient mutant), which formed less biofilm than the wild‐type strain (Liu *et al.*, 2001; Kang *et al.*, 2002). It should be clarified that both the wild‐type strain and mutants did not form pellicles at the air–liquid interface even though they were cultured in polystyrene microtitre plates without shaking overnight (data not shown), confirming that these observed biofilms are due to surface adhesion.

To investigate the impact of TapV on adherence to host cells, roots of hydroponically cultured tomato plants were dipped into bacterial suspension and adherent cells were quantified by dilution plating. Although the *tapV* mutant exhibited similar ability to the wild‐type strain to adhere to tomato roots at 16 hr post‐inoculation (hpi), its ability to adhere to tomato roots was significantly impaired during 20–24 hpi, which is consistent with the *pilA* mutant (a known T4P‐deficient mutant) exhibiting significantly impaired ability to adhere to tomato roots 16–24 hpi compared to the wild‐type strain (Figure 2c). All of these results confirm that TapV, but not Rsp0189, is important for T4P‐dependent swimming motility and root adherence, while TapV negatively affects biofilm information in *R. solanacearum*.

### 2.4 Expression of the T3SS is dependent on TapV both in vitro and in planta, but is T4P independent

**FIGURE 3:**
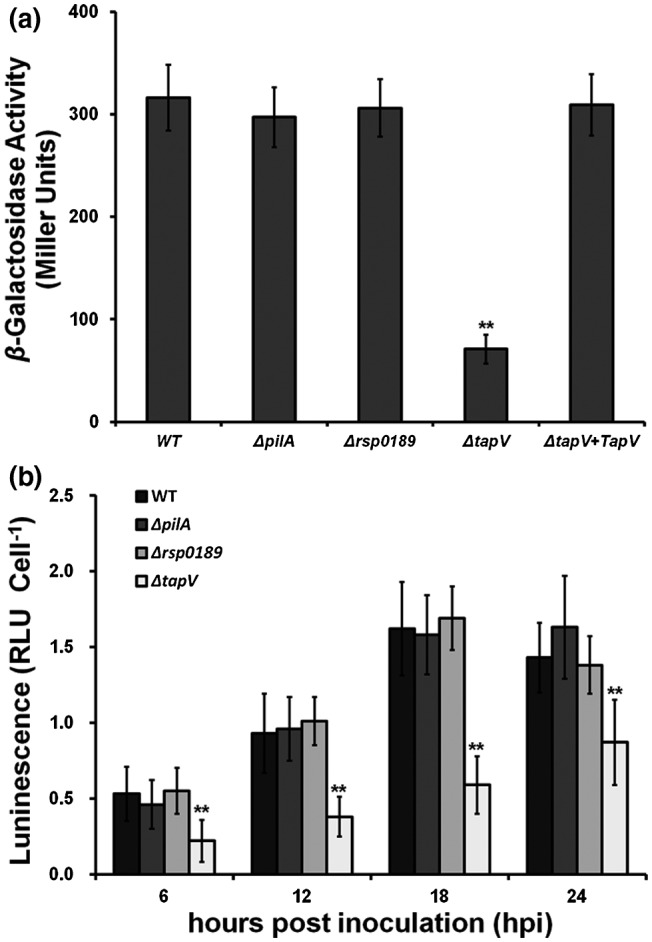
Expression of *popA* in *Ralstonia solanacearum* strains (a) in *hrp*‐inducing medium and (b) in planta (tobacco leaves). Wild‐type (WT), RK5050 (OE1‐1, *popA‐lacZYA*); Δ*pilA*, RQ6055 (RK5050, Δ*pilA*); Δ*rsp0189*, RQ6069 (RK5050, Δ*rsp0189*); Δ*tapV*, RQ5703 (RK5050, Δ*tapV*); Δ*tapV* + *tapV*, RQC0343 (RQ5703 with complementary *tapV*). Enzymatic activities in *hrp*‐inducing medium were carried out with the in vitro enzyme assay and presented in Miller units. In brief, cells were grown in *hrp*‐inducing medium to an OD_600_ of approximately 0.1 and subjected to the enzyme assay. Enzymatic activities in planta were carried out with the in planta enzyme assay and presented with luminescence normalized with cell numbers. In brief, tobacco leaves were infiltrated with a bacterial suspension of 0.1 OD_600_ and leaf disks were punched every 6 hr for the enzyme assay with a Galacto‐Light Plus kit. Cell population was quantified by dilution plating and luminescence was determined using a GloMax20 luminometer (Promega). Each assay was repeated with at least four biological replicates, including four replications per trial*.* The mean values of all experiments were averaged with *SD*, and the statistical significance between the WT strain RK5050 and mutants (*tapV* mutant RQ5703, *pilA* mutant RQ6055, or *rsp0189* mutant RQ6069) was assessed using a post hoc Dunnett test following analysis of variance. Significance level, **p < .01

TapV was originally screened as one of the T3SS‐regulating candidates by transposon mutagenesis, in which the expression profile of T3SS in *R. solanacearum* was monitored by *popA‐lacZYA* fusion (Zhang *et al.*, 2013). The *popA* gene is located upstream of the *hrp* regulon that belongs to T3Es and is directly controlled by HrpB. The *popA‐lacZYA* fusion exhibits an identical expression prolife to the *hrp* regulon under different conditions and this fusion does not affect the infection process of OE1‐1 toward host plants (Zhang *et al.*, 2013). The T3SS is not expressed in rich medium but is induced in *hrp*‐inducing medium (nutrient‐limited medium) (Cunnac *et al*., 2004; Yoshimochi *et al.*, 2009), and we assessed *popA* expression in *hrp*‐inducing medium. Consistent with those in transposon mutants, *popA* expression in the *tapV* mutant was significantly impaired in *hrp*‐inducing medium (71 versus 316 Miller units of the wild‐type reporter strain RK5050), and complementary *tapV* could completely restore impaired *popA* expression to that of RK5050 (Figure 3a). On the contrary, the *pilA* and *rsp0189* mutants exhibited similar expression levels of *popA* as RK5050 (Figure 3a), indicating that TapV, but not PilA and RSp0189, is required for T3SS expression in *R. solanacearum*. The requirement of TapV for T3SS expression is T4P independent.

Plant signals can greatly enhance T3SS expression to much higher levels compared with that in *hrp*‐inducing medium (Valls *et al.*, 2006; Yoshimochi *et al.*, 2009). We further investigated whether TapV is required for T3SS expression in planta. Tobacco leaves were infiltrated with a bacterial suspension at an OD_600_ of 0.1, and bacterial cells were recovered from infiltrated leaves at 6–24 hpi for the enzyme assay. Consistent with above result in *hrp*‐inducing medium, *popA* expression in tobacco leaves was significantly impaired with *tapV* deletion during 6–24 hpi, but not in the *pilA* or *rsp0189* mutants (Figure 3b), confirming that TapV is important for T3SS expression both in vitro and in planta, and the requirement of TapV for T3SS expression is T4P independent.

### 2.5 TapV greatly contributes to the pathogenicity of *R. solanacearum* in host plants

**FIGURE 4:**
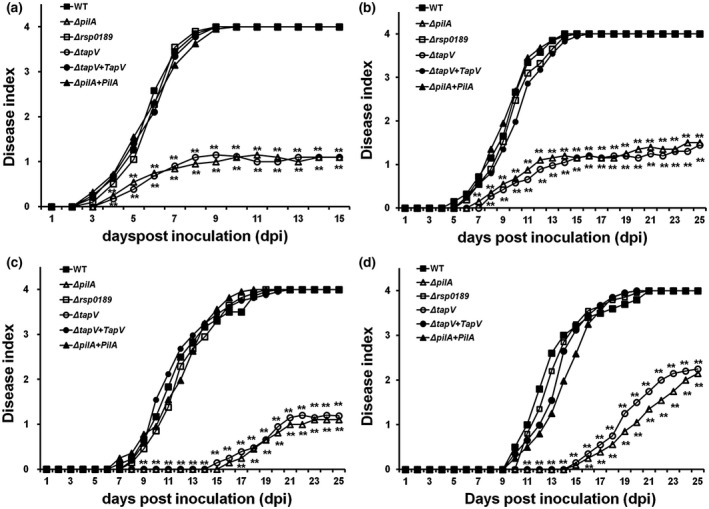
Virulence assay of *Ralstonia solanacearum* strains in (a) tomato plants with petiole inoculation, (b) tomato plants with soil‐soaking inoculation, (c) tobacco plants with leaf infiltration, and (d) tobacco plants with soil‐soaking inoculation. Wild‐type (WT), RK5050 (OE1‐1, *popA‐lacZYA*); Δ*pilA*, RQ6055 (RK5050, Δ*pilA*); Δ*rsp0189*, RQ6069 (RK5050, Δ*rsp0189*); Δ*tapV*, RQ5703 (RK5050, Δ*tapV*); Δ*tapV* + *tapV*, RQC0343 (RQ5703 with complementary *tapV*); Δ*pilA* + *pilA*, RQC0347 (RQ6055 with complementary *pilA*). For soil‐soaking inoculation, a bacterial suspension was poured into the pot soil of plants at a final concentration of 10^7^ cfu/g soil. For petiole inoculation, 3 µl of bacterial suspension at 10^8^ cfu/ml was dropped onto the freshly cut surface of petioles. For leaf infiltration, about 50 µl of bacterial suspension at 10^8^ cfu/ml was infiltrated into tobacco leaves with a blunt‐end syringe. Wilt symptoms were inspected daily and scored on a disease index scale from 0 to 4 (0, no wilting; 1, 1%–25% wilting; 2, 26%–50% wilting; 3, 51%–75% wilting; 4, 76%–100% wilted or dead). Each assay was repeated with at least four biological replicates, including 12 plants per trial. The mean values of all experiments were averaged and the statistical significance between the WT strain RK5050 and mutants (*tapV* mutant RQ5703 or *pilA* mutant RQ6055) was assessed using a post hoc Dunnett test following analysis of variance. Significance level, **p *<* .01

Both the T3SS and T4P play important roles in the pathogenicity of *R. solanacearum*, so we decided to evaluate whether TapV and Rsp0189 contribute to the pathogenicity of *R. solanacearum* toward host plants. Two host plants of tomato and tobacco plants were subjected to virulence assay with inoculation methods of soil‐soaking, which mimics natural invasion through roots, and petiole inoculation, which enables direct invasion into xylem vessels. Note that tobacco plants were inoculated with leaf infiltration, which enables direct invasion into intercellular spaces of tobacco leaves. RK5050 (the wild‐type strain) eventually killed all test plants, while the *tapV* mutant was significantly less virulent than RK5050, which killed only approximately 25% of test plants to 25 days post‐inoculation (dpi) regardless of different host plants and inoculation methods. Complementary *tapV* completely restored its virulence to that of the wild‐type strain in both tomato and tobacco plants (Figure 4a–d), confirming that TapV greatly contributes to the pathogenicity of *R. solanacearum* in host plants.

The virulence of the *pilA* and *rsp0189* mutants was also assessed in tomato and tobacco plants. Only the *pilA* mutant exhibited similar pathogenicity to the *tapV* mutant, which was significantly less virulent than RK5050 in test plants regardless of different host plants and inoculation methods. This is consistent with previous reports on some known *R. solanacearum* T4P‐deficient mutants (Liu *et al.*, 2001; Kang *et al.*, 2002), while the *rsp0189* mutant exhibited similar pathogenicity to RK5050 (Figure 4a–d). It is worthwhile noting that the *tapV* and *pilA* mutants exhibited almost equivalently reduced virulence compared with RK5050 in tomato and tobacco plants regardless of inoculation method.

### 2.6 TapV positively affects T3SS expression via the PhcA–TapV–PrhG–HrpB pathway

**FIGURE 5:**
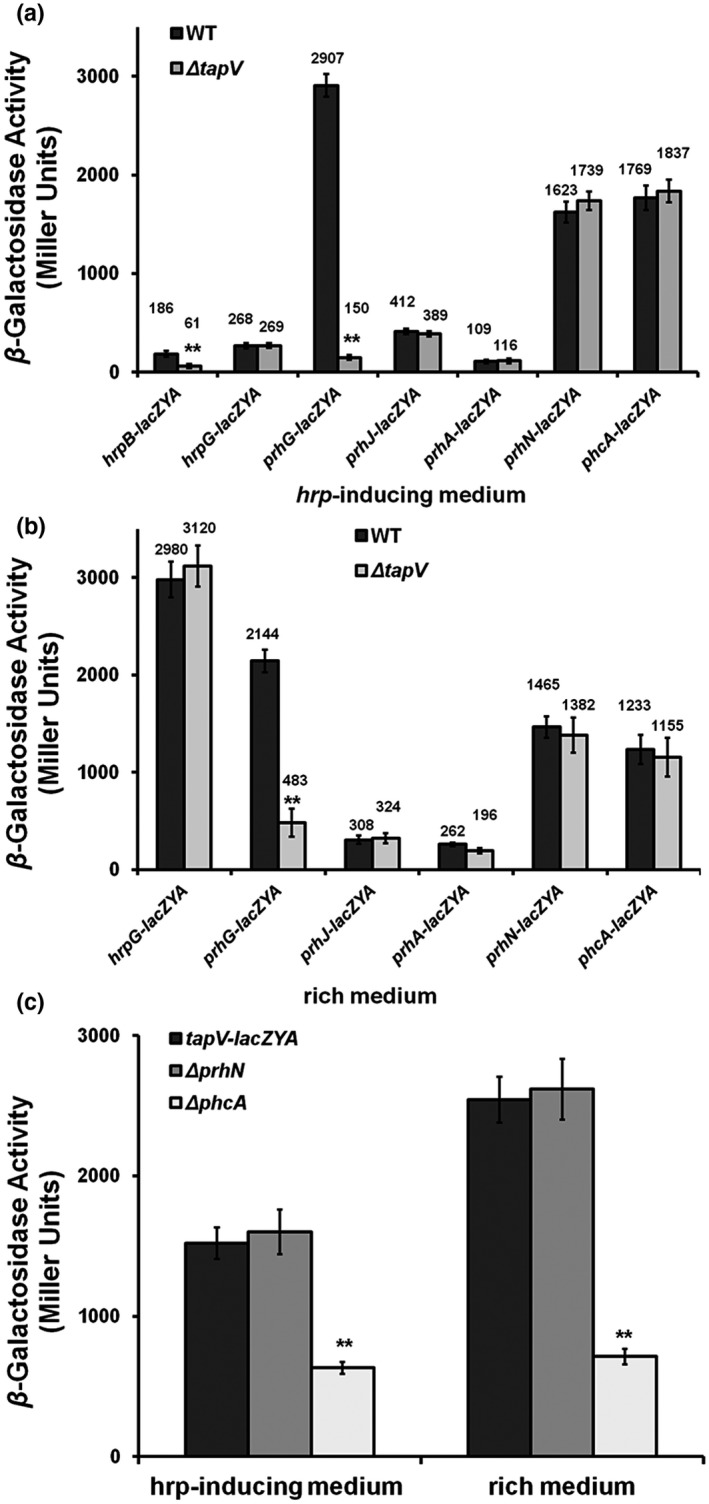
Effect pathway of TapV on the type III secretion system (T3SS). (a) and (b) Effect of *tapV* deletion on expression of a subset of regulatory genes in *hrp*‐inducing medium (a) and nutrient‐rich medium (b). Expression of *hrpB* and the T3SS was not expressed in rich medium, but induced in *hrp*‐inducing medium, and the *hrpB* expression was assessed in *hrp*‐inducing medium (a). The Δ*tapV* refers to deletion of *tapV* from each reporter strain of RK5046 (*hrpB‐lacZYA*), RK5120 (*hrpG‐lacZAY*), RK5212 (*prhG‐lacZAY*), RK5124 (*prhJ‐lacZAY*), RK5134 (*prhA‐lacZAY*), RK5619 (*prhN‐lacZAY*), and RK5043 (*phcA‐lacZAY*). (c) Expression of *tapV‐lacZYA* with deletion of *phcA* or *prhN*. The Δ*phcA* and Δ*prhN* refer to deletion of *phcA* or *prhN* from the reporter strain of RQC0341 (OE1‐1, *tapV‐lacZYA*), respectively. Cells were grown in *hrp*‐inducing medium or nutrient‐rich medium to an OD_600_ of about 0.1 and subjected to the β‐galactosidase assay (the in vitro enzyme assay). The mean values from all three biological replicates were averaged and are presented with *SD* (error bars). The statistical significance between parent strains (WT) and *tapV* mutants (Δ*tapV*) was assessed using a post hoc Dunnett test following analysis of variance. Significance level, **p *<* .01

In *R. solanacearum*, the T3SS is directly controlled by a master regulator HrpB, and *hrpB* expression is positively regulated by two close paralogs of HrpG and PrhG in a parallel way. Expression of *hrpG* is positively regulated by a signalling cascade of PrhA–PrhIR–PrhJ, while expression of *prhG* is independent of this cascade, which is positively regulated by PrhN and PhcA, a global regulator (Genin, 2010; Hikichi *et al.*, 2017). We generated *tapV* mutants from reporter strains of RK5046 (*hrpB‐lacZYA*), RK5120 (*hrpG‐lacZAY*), RK5212 (*prhG‐lacZAY*), RK5124 (*prhJ‐lacZAY*), RK5134 (*prhA‐lacZAY*), RK5619 (*prhN‐lacZAY*), and RK5043 (*phcA‐lacZAY*) to ascertain how TapV affects T3SS expression. Expression of *hrpB* and the T3SS was not activated in rich medium but was induced in *hrp*‐inducing medium (Cunnac *et al*., 2004; Yoshimochi *et al.*, 2009), and we assessed *hrpB* expression in *hrp*‐inducing medium. Deletion of *tapV* substantially impaired *hrpB* expression in *hrp*‐inducing medium (61 vs. 186 Miller units of RK5046 [*hrpB‐lacZYA*]) (Figure 5a), confirming that the impact of TapV on T3SS is mediated through the master regulator HrpB (Figure 5a). Furthermore, deletion of *tapV* substantially decreased *prhG* expression in both *hrp*‐inducing and rich media (150 vs. 2,907 Miller units of RK5212 [*prhG‐lacZAY*] in *hrp*‐inducing medium and 483 vs. 2,144 Miller units of RK5212 in rich medium), but there was no alteration on *hrpG* expression with *tapV* deletion in either medium (Figure 5a,b). Expression of *hrpG* is known to be regulated by the signalling cascade of PrhA–PrhIR–PrhJ–HrpG, while deletion of *tapV* did not affect expression of *prhA* and *prhJ* in either medium (Figure 5a,b), confirming that the impact of TapV on *hrpB* expression is mediated through PrhG, but independent of the signalling cascade of PrhA–PrhIR–PrhJ–HrpG.

We recently demonstrated that PhcA and PrhN positively regulate *prhG* expression (Zhang *et al.*, 2013, 2015), and we evaluated whether TapV affects their expression. Deletion of *tapV* did not affect expression of *prhN* and *phcA* in either medium (Figure 5a,b), and we turned to evaluate whether PhcA and PrhN affect *tapV* expression. A *tapV‐lacZYA* reporter fusion was generated and integrated into chromosome of OE1‐1 (the wild‐type strain) with the Tn*7*‐based chromosomal integration system to generate the reporter strain RQC0341. Note that integration of *tapV‐lacZYA* did not affect the basal growth rate of each mutant (data not shown). Deletion of *phcA* substantially impaired *tapV* expression in both rich and *hrp*‐inducing media (713 vs. 2,524 Miller units of RQC0341 in rich medium and 633 vs. 1519 Miller units of RQC0341 in *hrp*‐inducing medium) (Figure 5c), indicating that expression of *tapV* is positively regulated by PhcA. This is consistent with previous reports that the Phc confinement‐sensing system positively controls *pilA* expression in *R. solanacearum* (Kang *et al.*, 2002), whereas deletion of *prhN* did not affect *tapV* expression in either medium (Figure 5c), indicating that both TapV and PrhN positively regulate *prhG* expression in a parallel way. All of these results suggest that TapV positively affects T3SS expression via the PhcA–TapV–PrhG–HrpB pathway.

### 2.7 TapV is important for dispersal in host plants, but not for in planta proliferation

**FIGURE 6:**
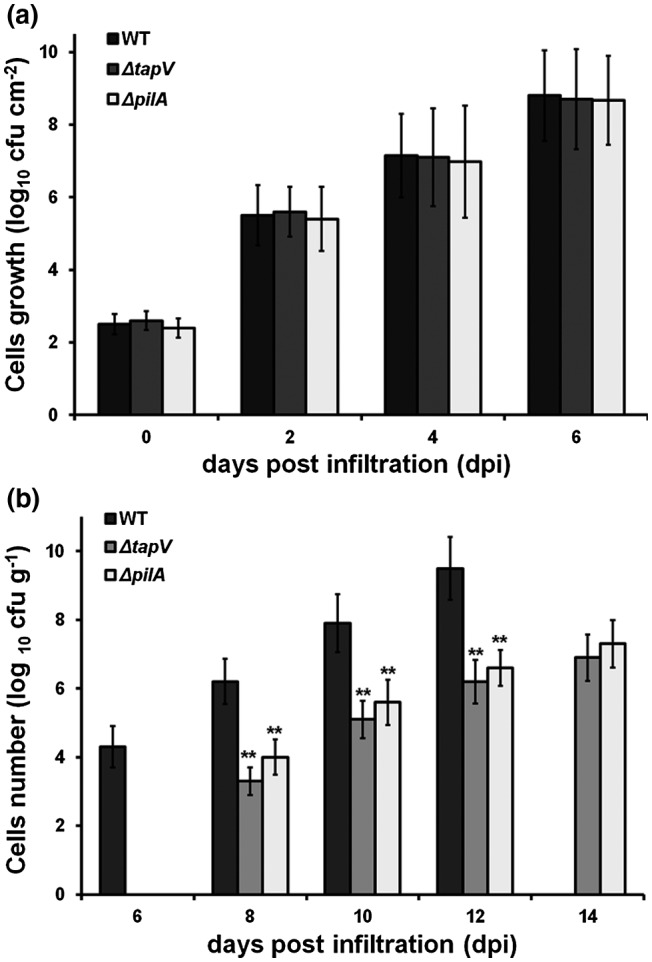
Bacterial proliferation and dispersal of *Ralstonia solanacearum* strains in tobacco plants. (a) Proliferation in intercellular spaces of tobacco leaves and (b) migration into petioles from infiltrated leaves. Wild‐type , RK5050 (OE1‐1, *popA‐lacZYA*); Δ*tapV*, RQ5703 (RK5050, Δ*tapV*); Δ*pilA*, RQ6055 (RK5050, Δ*pilA*). For proliferation in tobacco leaves (intercellular spaces), which is presented in log_10_ cfu/cm^2^, bacterial suspension at 10^4^ cfu/ml was infiltrated into tobacco leaves and leaf disks were punched every other day and subjected to quantification of cell densities by dilution plating. Leaf disks were punched to 6 days post‐infiltration (dpi), when infiltrated tobacco leaves became withered and dried. For migration into petioles from infiltrated leaves, which is presented in log_10_ cfu/g, about 50 μl of bacterial suspension at 10^8^ cfu/ml was infiltrated into tobacco leaves and petioles of the infiltrated leaves were periodically harvested for quantification of cells densities. The RK5050‐inoculated petioles were harvested until 12 dpi, when tobacco petioles became withered and fallen, while petioles inoculated with *tapV* or *pilA* mutants were harvested until 14 dpi, when tobacco plants started slightly wilting, but remained green. Each assay was repeated with at least four biological replicates, including four replicates per trial. The mean values from all experiments were averaged and are presented with *SD* (error bars). The statistical significance between the wild‐type strain (RK5050) and mutants (*tapV* mutant RQ5703 or *pilA* mutant RQ6055) was assessed using a post hoc Dunnett test following analysis of variance. Significance level, **p *<* .01

Extensive proliferation in host plants is one of the most important pathogenicity determinants of *R. solanacearum*. We thus assessed whether TapV is required for in planta proliferation. Tobacco leaves were infiltrated with bacterial suspension at a concentration of 10^4^ cfu/ml and cell growth in tobacco leaves was assessed every other day until 6 dpi, when tobacco leaves became withered and dried. Both the wild‐type strain and the *tapV* mutant exhibited similar proliferation in tobacco leaves from 2 to 6 dpi (Figure 6a), indicating that TapV is not required for intercellular proliferation of *R. solanacearum*.

Because the *tapV* mutant exhibited significantly less virulence than RK5050 in tobacco plants, we next assessed whether its impaired virulence was due to deficiency in migration from infiltrated leaves into petioles. Tobacco leaves were infiltrated with bacterial suspension at a concentration of 10^8^ cfu/ml and the cell number in petioles of the infiltrated leaves was measured every other day from 6 dpi, when RK5050 migrated into petioles, but not the *tapV* and *pilA* mutants, which could not be detected in petioles until 8 dpi. RK5050 could not be detected in petioles until 6 dpi with a density of approximately 2 × 10^4^ cfu/g, and grew extensively to a maximum of approximately 4 × 10^9^ cfu/g at 12 dpi, when petioles of infiltrated leaves became withered and dried (Figure 6b). The *tapV* and *pilA* mutants could not be detected in petioles of infiltrated leaves until 8 dpi with a densities of approximately 3 × 10^3^ and 10^4^ cfu/g, respectively, which is about three orders of magnitude less than RK5050 (Figure 6b), indicating that the *tapV* and *pilA* mutants are strikingly deficient in migration from infiltrated leaves into petioles. Compared to RK5050, the *tapV* mutant grew much more slowly, reaching a density of approximately 10^7^ cfu/g at 14 dpi, when infiltrated tobacco leaves became withered, while the tobacco plants were still green (Figure 6b). Note that the RK5050‐inoculated tobacco petioles became withered and fallen, and we did not assess bacterial growth in these petioles at 14 dpi (Figure 6b). The *pilA* mutant exhibited similar growth patterns to the *tapV* mutant in tobacco petioles of infiltrated leaves (Figure 6b). All of this confirmed that TapV is important for *R. solanacearum* to migrate from intercellular spaces into host petioles, which might be T4P dependent.

**FIGURE 7:**
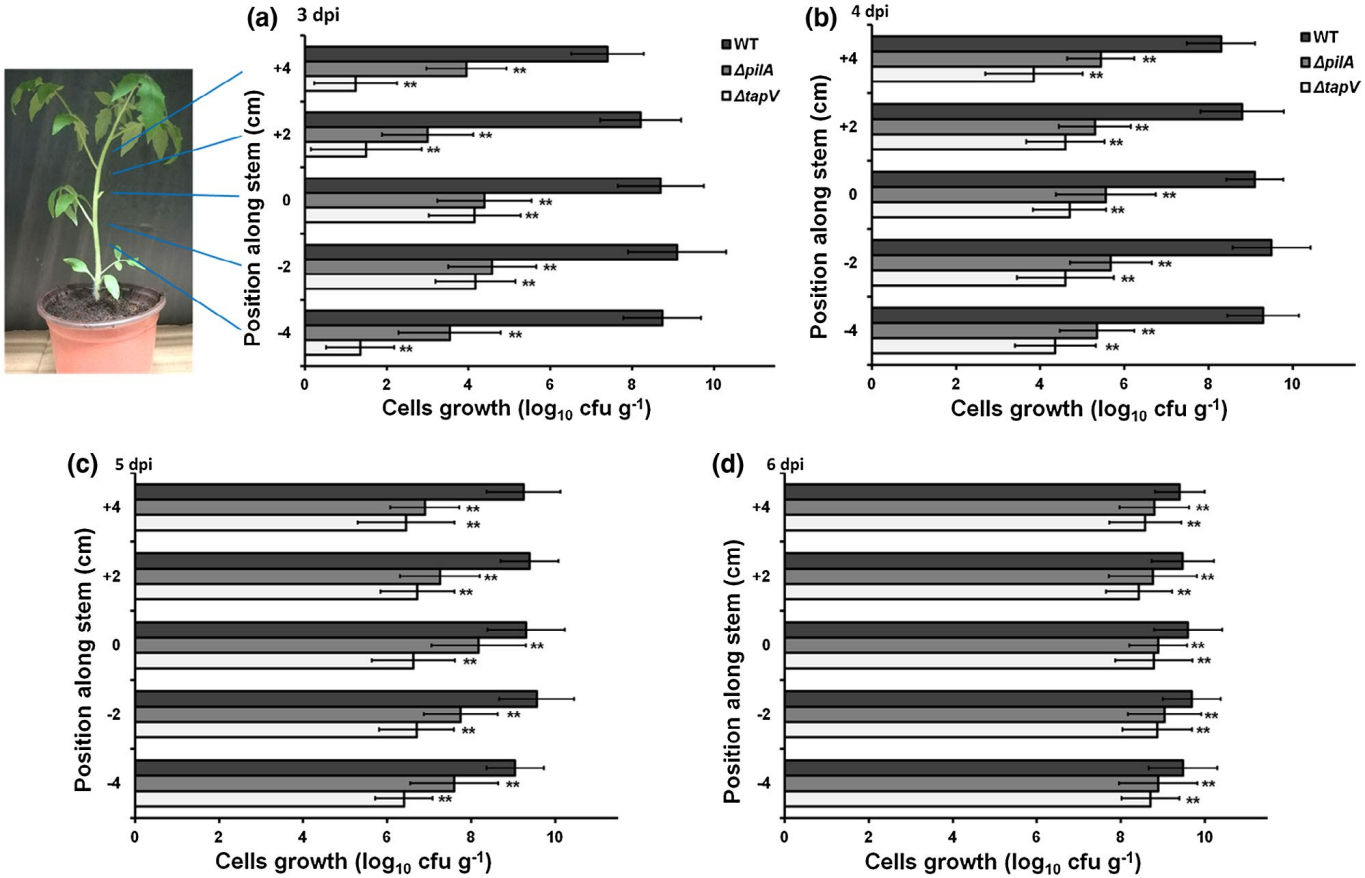
Bacterial dispersal of *Ralstonia solanacearum* strains in tomato xylem vessels. wild‐type (WT), RK5050 (OE1‐1, *popA‐lacZYA*); Δ*tapV*, RQ5703 (RK5050, Δ*tapV*); Δ*pilA*, RQ6055 (RK5050, Δ*pilA*). About 3‐week‐old tomato plants were inoculated by dropping 2 µl of bacterial suspension at a density of 10^6^ cfu/ml onto the freshly cut surface of petioles. Tomato stems including the inoculation site were periodically harvested and cut into sections 2 cm in length. Stem sections around the inoculation site were set as position 0, and those 2 cm above and below position 0 were set as positions +2 and −2, and then positions +4 and −4. Cell densities in each stem section were quantified with dilution plating and presented in log_10_ cfu/g, which was assessed at 3 days post‐inoculation (dpi), when RK5050‐inoculated tomato plants started wilting, and continued to be assessed on a daily basis for days 4, 5, and 6, when RK5050‐inoculated tomato plants became wilted and died at 6 dpi. Each assay was carried out with at least four biological replicates, including four replicates per trial. The mean values from all experiments were averaged and are presented with *SD* (error bars). The statistical significance between the WT strain RK5050 and mutants (*tapV* mutant RQ5703 or *pilA* mutant RQ6055) was assessed using a post hoc Dunnett test following analysis of variance. Significance level, **p *<* .01

The *tapV* mutant exhibited significantly less virulence in petiole‐inoculated tomato plants, by which *R. solanacearum* migrated into xylem vessels from tomato petioles. We further assessed the requirement of TapV for migration of *R. solanacearum* from inoculated tomato petioles into xylem vessels as shown in Figure 7. In brief, tomato plants were inoculated by dropping 2 µl of bacteria suspension onto the fresh‐cut surface of petioles, and stems were periodically harvested for quantification of cell densities by dilution plating. Tomato stems were cut into sections 2 cm in length and stem sections (2 cm) around the inoculation site were set as position 0, then those 2 cm above and below position 0 were set as positions +2 and −2, respectively, then positions +4 and −4. Cell densities in stem sections were quantified at 3 dpi, when RK5050‐inoculated tomato plants started wilting, and continued to be assessed on a daily basis for days 4, 5, and 6, when RK5050‐inoculated tomato plants became wilted and died at 6 dpi (Figure 7b–d). RK5050 (the wild‐type strain) grew well in tomato stems at 3 dpi, reaching a density of approximately 10^9^ cfu/g in stem sections at positions 0, −2, and −4 (below the inoculation site), and 10^7^–10^8^ cfu/g in stem sections at positions +2 and +4 (above the inoculation site) (Figure 7a). At 3 dpi, the *tapV* mutant grew slowly to a density of approximately 10^4^ cfu/g in stem sections at positions 0 and −2 (below the inoculation site), while it was faintly detected in stem sections at positions +2, +4, and −4 with a density of less than 10^2^ cfu/g (Figure 7a). RK5050 grew quickly in stems and reached to a maximum of approximately 10^10^ cfu/g at 4–6 dpi, while the *tapV* mutant exhibited significantly impaired proliferation in different stem sections compared to RK5050 (Figure 7b–d). For instance, the *tapV* mutant grew to a density of approximately 5 × 10^4^ cfu/g in stem sections at positions 0, −2, and +2 (around the region of 2 cm above and below the inoculated site) at 4 dpi, but approximately 10^4^ cfu/g in stem sections at positions +4 and −4 (Figure 7b). The *tapV* mutant grew to a density of approximately 10^6^ cfu/g in all stem sections (positions 0, −2, −4, +2, and +4) at 5 dpi (Figure 7c) and reached a maximum of approximately 10^9^ cfu/g in all stem sections at 6 dpi (Figure 7d), all of which were significantly less than those of RK5050 at the corresponding positions. Migration and proliferation of the *pilA* mutant in stem sections was also significantly impaired compared to that of the wild‐type strain, but much higher than for the *tpaV* mutant at 3–5 dpi (Figure 7b–d). These results confirm that TapV plays an important role in the migration of *R. solanacearum* from petioles into host xylem vessels.

### 2.8 TapV is important for expression of a subset of T3Es but not for elicitation of the hypersensitive response

Expression of abundant T3Es is directly controlled by HrpB, which is significantly impaired in the *tapV* mutant. We thus assessed whether expression of T3Es was impaired with *tapV* deletion. In this study, a total of 11 T3Es, including RipAA, RipAR, RipB, RipD, RipE1, RipO, RipP1, RipR, RipTAL, RipW, and RipX (PopA as positive control), were selected for quantification of message RNA levels with quantitative reverse transcription PCR (RT‐qPCR). Expression levels of these T3Es were significantly reduced in the *tapV* mutant (Figure S1), confirming that TapV is important for expression of a subset of T3Es in *R. solanacearum.*

Several T3Es, such as RipAA and RipP1, have been experimentally validated to be responsible for hypersensitive response (HR) elicitation of *R. solanacearum* GMI1000 in tobacco leaves (Poueymiro *et al.*, 2009; Peeters *et al.*, 2013), so we assessed the requirement of TapV for HR elicitation of GMI1000 in tobacco leaves. Tobacco leaves were infiltrated with a cell suspension at OD_600_ of 0.1 and symptom development of necrotic lesions was investigated. The *tapV* (GF0067), *pilA* (GF0106), and *rsp0189* mutants (GF0108) exhibited similar HR development to the parent strain GMI1000 in tobacco leaves (Figure S2), indicating that TapV, PilA, and Rsp0189 are not required for the HR elicitation of GMI1000 in tobacco leaves.

## 3 DISCUSSION

In the present study, we provided multiple lines of evidence to demonstrate that TapV is a novel essential component for T4P functions in *R. solanacearum*. Although functions of T4P are diverse in different bacteria, T4P have been generally demonstrated to be important for twitching motility, biofilm formation, adhesion to host cells, DNA uptake, and bacteriophage infection (Burdman *et al.*, 2011; Dunger *et al.*, 2016). Twitching motility is one of the most representative T4P‐dependent properties in many bacteria, and is confirmed to be abolished in T4P‐deficient mutants (*pilA* mutants) in *R. solanacearum* (Liu *et al.*, 2001; Kang *et al.*, 2002; Wairuri *et al.*, 2012). TapV contains the typical LysM domain, PTR domain, and transmembrane domain. Several proteins containing the LysM domain and PTR domain have been validated to be required for T4P assembly in different bacterial species that bind to peptidoglycan and associate strongly with components of T4P on the outer membrane (Bateman and Bycroft, 2000; Wehbi *et al.*, 2011; Sieweringa *et al*., 2014). The *tapV* mutant exhibited the same phenotype as the *pilA* mutant on twitching motility, confirming that TapV is required for T4P assembly. TapV might bind to peptidoglycan and associate with some components of T4P on the outer membrane in *R. solanacearum*. Moreover, deletion of *tapV* significantly impaired the ability of *R. solanacearum* to adhere to host roots, migrate in host plants, and swim on the soft‐agar surface, similarly to the phenotypes of the *pilA* mutant (T4P‐deficient mutant). All this provides strong evidence to support the fact that TapV plays an essential role in functional T4P in *R. solanacearum*, while Rsp0189, the homolog of TapV, was not required for T4P even though they share 54% of identity at N‐termini of about 300 amino acids containing the typical LysM domain (85% of identity at the typical LysM domain) and 82% of identity at the central region of 150 amino acids containing the TPR domain. The precise roles of TapV in T4P remain to be further elucidated in *R. solanacearum*, including how TapV interacts with T4P components to promote T4P assembly, and which residues or domains play important roles in the interaction between TapV and other T4P components.

Some T4P‐dependent properties are also important requisites for the virulence of many pathogenic bacteria of animals and plants (Burdman *et al.*, 2011; Dunger *et al.*, 2016). It is not surprising that deletion of *tapV* significantly impaired the pathogenicity of *R. solanacearum* toward host tomato and tobacco plants. In *R. solanacearum*, several reports have revealed that T4P mutants exhibited pathogenicity‐related phenotypes, including reduced autoaggregation, biofilm formation, and lack of attachment ability to tomato roots (Liu *et al.*, 2001; Kang *et al.*, 2002; Wairuri *et al.*, 2012). Although the *tapV* mutant proliferated normally as the wild‐type strain in the intercellular space of tobacco leaves where the bacterium was infiltrated, it exhibited a severely impaired ability to spread throughout host plants, including migration from the intercellular spaces of infiltrated leaves into petioles, and migration from petioles into xylem vessels of stems. Although the *tapV* mutant can eventually migrate into and extensively proliferate in xylem vessels of host plants, the growth rate of the *tapV* mutant in xylem vessels was significantly lower than that of the wild‐type strain. Moreover, the proliferation of the *tapV* mutant in xylem vessels and tobacco petioles was less than that of the wild‐type strain by more than one order of magnitude. We speculate that the impaired ability of the *tapV* mutant to spread and proliferate throughout xylem vessels is the main reason for its significantly impaired virulence. Given the fact that the *pilA* mutant, a known T4P‐deficient mutant in *R. solanacearum*, exhibited similar phenotypes to the *tapV* mutant in planta proliferation and migration throughout xylem vessels, the impaired ability of the *tapV* mutant in proliferation and migration might be due to theT4P deficiency. This is consistent with the fact that Rsp0189 is independent of functional T4P and hence is not required for pathogenicity of *R. solanacearum* toward host plants.

Substantial evidence has demonstrated that the ability to adhere to the host cell surface is important for bacteria to initiate infection, and twitching motility is important for biofilm formation, both of which allow bacteria to efficiently colonize different niches (O’Toole *et al.*, 2000; Chiang and Burrows, 2003; Kaiser, 2003; Klausen *et al.*, 2003). The *tapV* and *pilA* mutants exhibited significantly impaired swimming ability, which is known to make a most important contribution to bacterial wilt virulence, especially in the early stages of host plant invasion and colonization (Tans‐Kersten *et al*., 2004; Lowe‐Power *et al.*, 2018). It is consistent with our observation that the ability to adhere to tomato roots was significantly impaired in the *tapV* and *pilA* mutants. Given that the T4P makes its contribution to multiple stages of pathogenesis (Lowe‐Power *et al.*, 2018), and the *tapV* and *pilA* mutants exhibited significantly impaired twitching motility, one of the most representative T4P‐dependent features, significantly impaired migration and proliferation of the *tapV* mutant in host plants might be due to deficient T4P in *R. solanacearum*. Intriguingly, the *tapV* mutant produced much more biofilm than the wild‐type strain in polystyrene plates, which is different to many T4P‐deficient mutants (Burdman *et al.*, 2011; Dunger *et al.*, 2016). With observation by scanning electronic microscope, it was recently reported that *R. solanacearum* can produce mushroom‐type biofilms on the surface of tomato cells after invasion into intercellular spaces (Mori *et al.*, 2016, 2017). A similar observation also reported that twitching motility is required for development of biofilm mushroom‐like caps in *P.* *aeruginosa* (Klausen *et al.*, 2003). TapV might play different roles in biofilm formation under different conditions and sophisticated observation will be required to evaluate its impact on mushroom‐type biofilm in host plants.

It is interesting that TapV was found to positively affect T3SS expression, another essential pathogenicity determinant in *R. solanacearum* (Hikichi *et al.*, 2007; Genin, 2010). It is intriguing that the *pilA* mutant exhibited similar expression levels of the T3SS both in vitro and in planta, indicating that the requirement of TapV for T3SS expression is T4P independent. TapV might play a novel role in affecting T3SS expression in plant pathogenic bacteria. It has been reported that expression of some T3SS‐related genes was induced by bacterial contact to host cells mediated by T4P in *P. aeruginosa* (Yahr and Wolfgang, 2006). Expression of *hrpB* and T3SS is not activated until the bacterium comes into contact with host signals or some mimic signals (Marenda *et al.*, 1998; Yoshimochi *et al.*, 2009; Zhang *et al.*, 2013). It was previously reported that a *R. solanacearum* GMI1000 *hrpY* mutant, deficient in contact with host cells with *hrp* pili, can adhere to suspension‐cultured host cells and induce T3SS expression as the wild‐type strain (Aldon *et al*., 2000; VanGijsegem *et al.*, 2000). The T4P‐dependent adhesion might enable *R. solanacearum* to make close contact with host cells, resulting in the induction of T3SS expression. HrpG and PrhG, two close paralogs of TCS response regulators, can respond to host signals and activate *hrpB* expression in a parallel way (Plener *et al.*, 2010; Zhang *et al.*, 2013). We previously reported that *prhG* expression was positively controlled by a global regulator PhcA, a central regulator in the global regulation network of pathogenicity that controls multiple genes directly or indirectly in *R. solanacearum* (Schneider *et al*., 2009; Genin and Denny, 2012; Zhang *et al.*, 2013). Consistent with previous reports that *pilA* expression at the exponential phase of growth is positively controlled by PhcA in *R. solanacearum* (Kang *et al.*, 2002), *tapV* expression was revealed to be positively regulated by PhcA, indicating that the impact of T4P on the T3SS is also mediated via PhcA. We further revealed that impact of TapV on the T3SS is mediated through the PhcA‐TapV‐PrhG‐HrpB pathway, but independent of the PrhA‐PrhIR‐PrhJ‐HrpG‐HrpB signalling cascade, whereas no interaction was detected between expression of *tapV* and *prhN*, both of which might positively regulate *prhG* expression in a parallel way.

Expression levels of *hrpB* and a subset of T3Es were significantly impaired in *tapV* mutants, which is consistent with the fact that the master regulator HrpB directly controls entire T3SS and T3Es (Valls *et al.*, 2006; Hikichi *et al.*, 2007). The *hrp*B mutant is well known to be indisposed to proliferate in host plants and completely loses pathogenicity in host plants (Valls *et al.*, 2006; Yoshimochi *et al.*, 2009), while the *tapV* mutant exhibited equal proliferation to the wild‐type strain in the intercellular spaces of tobacco leaves, indicating that weakly expressed *hrpB* might be enough to fulfil bacterial proliferation in host plants. This is consistent with our observation that HR elicitation of GMI1000 in tobacco leaves is not altered with *tapV* deletion. Note that expression of a subset of T3Es was substantially impaired in *hrp*‐inducing medium, but not diminished, and expression of *ripX* (*popA*, one of the T3Es) in tobacco leaves remained at about a quarter to one third of expression levels of the wild‐type strain. These weakly expressed T3Es might be enough to be recognized by tobacco plants and then trigger plant immunity. This is consistent with our previous reports that several T3Es substantially decreased mutants exhibiting identical HR elicitation as GMI1000 in tobacco leaves (Zhang *et al.*, 2018).

In summary, our genetic results demonstrated TapV as a novel essential component for functional T4P in *R. solanacearum* that greatly contributes to the infection process in host plants. TapV plays important roles in the T4P‐dependent properties of twitching motility, swimming motility, and host cell adhesion, and hence the *tapV* mutant is deficient in dispersal in host plants. Moreover, it was revealed that TapV positively affects the T3SS expression both in vitro and in planta through the PhcA‐TapV‐PrhG‐HrpB pathway. This is the first report that T4P components affect T3SS expression, providing a novel insight into understanding the various biological functions of T4P and the complex regulatory pathway in T3SS in *R. solanacearum*.

## 4 MATERIALS AND METHODS

### 4.1 Bacterial strains and growth conditions

*Escherichia coli* strains DH12S and S17‐1 were grown at 37 °C in Luria Bertani medium for plasmid construction and conjugational transfer, respectively. *R. solanacearum* strains were grown at 28 °C in nutrient‐rich medium (broth medium) or nutrient‐limited medium (*hrp‐*inducing medium, sucrose medium) (Yoshimochi *et al.*, 2009). The *R. solanacearum* strains used in this study are listed in Table 1. They are derivatives of GMI1000 and OE1‐1. OE1‐1 is virulent in tomato and tobacco plants (Kanda *et al.*, 2003), while GMI1000 is avirulent in tobacco plants and elicits a hypersensitive response in tobacco leaves (Poueymiro *et al.*, 2009).

**TABLE 1:** Bacterial strains used in this study

Strain	Relative characteristics	References
OE1‐1	Wild‐type, race 1, biovar 3	Kanda *et al.* (2003)
RK5046	OE1‐1, *hrpB‐lacZYA*	Yoshimochi *et al.* (2009)
RK5050	OE1‐1, *popA‐lacZYA*	Yoshimochi *et al.* (2009)
RK5120	OE1‐1, *hrpG‐lacZYA*	Yoshimochi *et al.* (2009)
RK5212	OE1‐1, *prhG‐lacZYA*	Zhang *et al.* (2013)
RQ5703	RK5050, Δ*tapV*	This study
RQ6055	RK5050, Δ*pilA*	This study
RQ6069	RK5050, Δ*rsp0189*	This study
RQC0343	RQ5703, *tapV* complementation	This study
RQC0347	RQ6055, *pilA* complementation	This study
RQ5720	RK5046, Δ*tapV*	This study
RQ5954	RK5120, Δ*tapV*	This study
RQ6040	RK5212, Δ*tapV*	This study
RQC0341	OE1‐1, *tapV‐lacZYA*	This study
RQC0342	OE1‐1, *tapV‐lacZYA*, Δ*phcA*	This study
GMI1000	Wild‐type, race 1, biovar 4	Salanoubat *et al*. (2002)
GF0067	GMI1000, Δ*tapV*	This study
GF0106	GMI1000, Δ*pilA*	This study
GF0108	GMI1000, Δ*rsp0189*	This study

### 4.2 Mutant generation with in‐frame deletion of *tapV*, *pilA,* and *rsp0189*

Mutants with in‐frame deletion of target genes were generated with pK18mobsacB‐based homologous recombination as described previously (Zhang *et al.*, 2015). In general, two DNA fragments flanking target genes were conjugated with joint PCR and cloned into pK18mobsacB to generate pK18dtapV, pK18dpilA, and pK18d0189, which were subjected  to in‐frame deletion of genes *tapV*, *pilA*, and *rsp0189*, respectively. After validating sequences, each plasmid was individually transferred into *R. solanacearum* by conjugation with S17‐1, and desired the mutants (listed in Table 1) were generated and confirmed by colony PCR with respective primer pairs (listed in Table S1).

### 4.3 Complementation analyses

Genetic complementation was performed with the pUC18‐mini‐Tn*7*T‐Gm based site‐specific chromosomal integration system (Tn*7* insertion) as described previously (Choi *et al.*, 2005; Zhang *et al.*, 2011). In general, a DNA fragment, containing the target gene and an upstream region of about 600 bp, empirically harbouring a native promoter, was PCR amplified and cloned into pUC18‐mini‐Tn*7*T‐Gm to generate pUCtapV and pUCpilA. After validating sequences, each complementary gene was integrated into the chromosome of corresponding mutants at a single attTn*7* site (25 bp downstream of *glmS*) and the desired mutants were generated and confirmed by colony PCR with a primer pair of glmsdown and Tn7R (Zhang *et al.*, 2011).

### 4.4 Construction of reporter fusion of *tapV‐laZYA* for promoter activity assay

Reporter strains with *tapV‐laZYA* fusion were generated with the Tn*7* insertion as described previously (Zhang *et al.*, 2015). In general, promoterless *lacZYA* was fused to *tapV* at 54 bp after the start codon, in which 6 bp of nucleotide acids were replaced in *Kpn*I by PCR for *lacZYA* insertion. The DNA fragment containing the promoter region (upstream region of about 600 bp) and the *Kpn*I site was first cloned into pUC18‐mini‐Tn*7*T‐Gm and then promoterless *lacZYA* was inserted to generate pUCtapV‐lacZYA. After validating the sequence, the *tapV‐laZYA* was integrated into chromosomes of different strains and the desired mutants were generated (Table 1).

### 4.5 β‐galactosidase assay

The β‐galactosidase assay was performed to evaluate expression levels of *lacZYA*‐fused genes both in vitro and in planta as described previously (Zhang *et al.*, 2013). Enzyme activity in vitro was expressed in Miller units (Miller, 1992), and that in planta was normalized with luminescence divided by cell number (Zhang *et al.*, 2013). Each assay was carried out with at least four biological replicates, and each trial included four replications. Mean values of all experiments were averaged with *SD*, and the statistical significance was assessed using a post hoc Dunnett test following analysis of variance (ANOVA).

### 4.6 Virulence assay and HR test

Virulence assay was carried out on wilt‐susceptible tomato plants (*Solanum lycopersicum* 'Moneymaker') and tobacco plants (*Nicotiana tabacum* 'Bright Yellow'), which were grown at 25 °C for 2–3 or 3–4 weeks, respectively, and subjected for virulence assay (Yao and Allen, 2007; Zhang *et al.*, 2013). Briefly, tomato plants were inoculated by the method of soil‐soaking inoculation that mimics natural invasion through roots, and petiole inoculation that enables direct invasion into xylems vessels. Tobacco plants were inoculated by methods of soil‐soaking and leaf‐infiltration that enable direct invasion into intercellular spaces (Zhang *et al.*, 2013). Each assay was carried out with at least four biological replicates and each trial included 12 plants. Wilt symptoms of plants were rated using a 1–4 disease index and the mean values of all experiments were averaged. The statistical significance was assessed using a post hoc Dunnett test following ANOVA.

The HR test was carried out in tobacco leaves of *N. tabacum* 'Bright Yellow' with leaf infiltration. In general, approximately 50 μl of bacterial suspension at a density of 10^8^ cfu/ml was infiltrated into tobacco leaves with a blunt‐end syringe and symptom development of necrotic lesions was recorded periodically (Zhang *et al.*, 2015). Each test was carried out with four biological replicates including four leaves per trial and a representative result was presented.

### 4.7 Observation of twitching motility

Twitching motility was observed as described previously (Turnbull and Whitchurch, 2014; Zhou *et al.*, 2015). In brief, 10 µl of bacterial suspension at an OD_600_ of 0.1 was dropped onto the surface of 1% broth agar on sterile slides, and kept at 28 °C for 24 hr with coverslips. The twitching zones were observed with a light microscope (Olympus CX21) equipped with a 40× objective. Each assay was carried out with three biological replicates, including four replications per trial. Twitching zones were observed on all samples and a representative result was presented.

### 4.8 Bacterial growth and dispersal *in planta*

Bacterial growth in planta was assessed as described previously (Zhang *et al.*, 2013). In brief, bacterial cells were collected daily from tobacco leaves, tobacco petioles, and tomato stems for quantification with dilution plating. Cell densities in leaves and petioles (stems) were expressed in log_10_ cfu/cm^2^ and log_10_ cfu/g, respectively.

The in planta dispersal was carried out in tobacco petioles and tomato stems, respectively. Briefly, approximately 50 μl of bacterial suspension at 10^8^ cfu/ml was infiltrated into tobacco leaves and the tobacco petioles of the infiltrated leaves were periodically harvested for quantification. For tomato petioles, 2 µl of bacterial suspension at a density of 10^6^ cfu/ml was dropped onto the fresh‐cut surface of tomato petioles. Tomato stems were harvested periodically and cut into sections of 2 cm in length. Stem sections (2 cm) around the inoculation site were set as position 0, and those 2 cm above and below position 0 were set as positions +2 and −2, respectively, and then positions +4 and −4. Cell densities in the stem sections were quantified with dilution plating and expressed in log_10_ cfu/g. Each assay was repeated for at least four biological replicates, including four replications per trial*.* Mean values of all experiments were averaged with *SD*, and the statistical significance was assessed using a post hoc Dunnett test following ANOVA.

### 4.9 Bacterial attachment assays

Bacterial attachment was carried out on tomato roots as described previously (Liu *et al.*, 2001). Briefly, the root systems of 3‐week‐old tomato plants with sterile culture were immersed in 10 ml of bacterial suspension at a density of 10^7^ cfu/ml and kept at 25 °C without agitation for 36 hr. Lateral roots were then periodically harvested, gently dipped in water twice to remove unattached bacteria, and the cell number was quantified with dilution plating. Each assay was repeated for at least four biological replicates, including four replications per trial*.* Mean values of all experiments were averaged with *SD*, and the statistical significance was assessed using a post hoc Dunnett test following ANOVA.

### 4.10 Biofilm formation and swimming motility assay

Biofilm formation was assessed in 96‐well polystyrene microtitre plates as described previously (Mori *et al.*, 2016; Zhang *et al.*, 2018). Briefly, 20 µl of bacterial suspension at an OD_600_ of 1.0 was inoculated into 180 µl of fresh broth medium and kept at 28 °C for 24 hr without shaking. After staining with crystal violet, biofilm formation was quantified by measuring absorbance at 530 nm (A_530_) and normalized with the cell number (OD_600_). The swimming motility assay was carried out on semisolid media (0.3% agar plates) as reported previously (Kelman and Hruschka, 1973). The diameters of the swimming halos on semisolid media (28 °C for 48 hr) were measured. Each assay was repeated independently for four biological replicates, including three replications per trial. The mean values of all experiments were averaged with *SD*, and the statistical significance was assessed using a post hoc Dunnett test following ANOVA.

### 4.11 RT‐qPCR analysis

Expression levels of genes without *lacZYA* fusion were quantified by RT‐qPCR analysis as described previously (Zhang *et al.*, 2018). Briefly, total RNA was isolated by the TRIzol reagent method (Life Technologies) and cDNA was synthesized using the PrimeScript RT Reagent Kit with gDNA Eraser (Takara). The One Step SYBR PrimeScript PLUS RT‐PCR Kit (Takara,) was used for RT‐qPCRs with the Applied Biosystems 7500 Real‐Time PCR System. The primers used in this study were selected as previously described. Among them the *serC* gene was selected as reference for normalization of gene expression, and *ripX* was selected as positive control (Monteiro *et al.*, 2012; Zhang *et al.*, 2018). Each assay was carried out from RNA isolation with three biological replicates and each trial included four replications. The mean values of all experiments were averaged with *SD*, and the statistical significance between the wild‐type strain and mutants was assessed using a post hoc Dunnett test following ANOVA.

## Supporting information


**FIGURE S1** Relative expression of T3Es genes in the *tapV* mutant. Strains were grown in *hrp‐*inducing medium to an OD_600_ of about 0.1 and total RNA was isolated. The cDNA was synthesized using the PrimeScript RT Reagent Kit with gDNA Eraser and message RNA levels of representative T3Es genes were determined by RT‐qPCR with reference gene as* serC* for normalization. Normalized values of *tapV* mutant were divided by those of wild‐type strain (WT) and relative values (relative expression) were presented. Mean values of at least three biological replicates were averaged and presented with *SD* (error bars). Statistical significance between the wild‐type strain and *prhP* mutants was assessed using a post hoc Dunnett test following ANOVA. Significance level, **p *<* .01

**FIGURE S2** HR test. Approximate 50 µl of bacterial suspension at 10^8^ cfu/ml was infiltrated into tobacco leaves with a blunt‐end syringe. (a) GMI1000, the wild‐type strain, (b) GF0067 (GMI1000, Δ*tapV*), (c) GF0106 (GMI1000, Δ*pilA*), (d) GF0108 (GMI1000, Δ*rsp0189*), and (e) distilled water. Development of necrotic lesions was observed periodically and pictures were taken. Each experiment was repeated at least for four times and each treatment contained four plants. The results presented are from a representative experiment, and similar results were obtained in all experiments

**TABLE S1** Primers used in this study

## Data Availability

The data that support the findings of this study are available from the corresponding author upon reasonable request.
