# Supplementary material for: Expression of Ralstonia solanacearum type III secretion system is dependent on a novel type 4 pili (T4P) assembly protein (TapV) but is T4P independent
Source: Mol Plant Pathol. 2020 Mar 20;21(6):777–93. doi: 10.1111/mpp.12930 (PMC7214476; doi:10.1111/mpp.12930)
Supplement: Supplementary file 1 — FIGURE S1 Relative expression of T3Es genes in the tapV mutant. Strains were grown in hrp‐inducing medium to an OD600 of about 0.1 and total RNA was isolated. The cDNA was synthesized using the PrimeScript RT Reagent Kit with gDNA Eraser and message RNA levels of representative T3Es genes were determined by RT‐qPCR with reference gene as serC for normalization. Normalized values of tapV mutant were divided by those of wild‐type strain (WT) and relative values (relative expression) were presented. Mean values of at least three biological replicates were averaged and presented with SD (error bars). Statistical significance between the wild‐type strain and prhP mutants was assessed using a post hoc Dunnett test following ANOVA. Significance level, **p < .01 [file MPP-21-777-s001.docx]

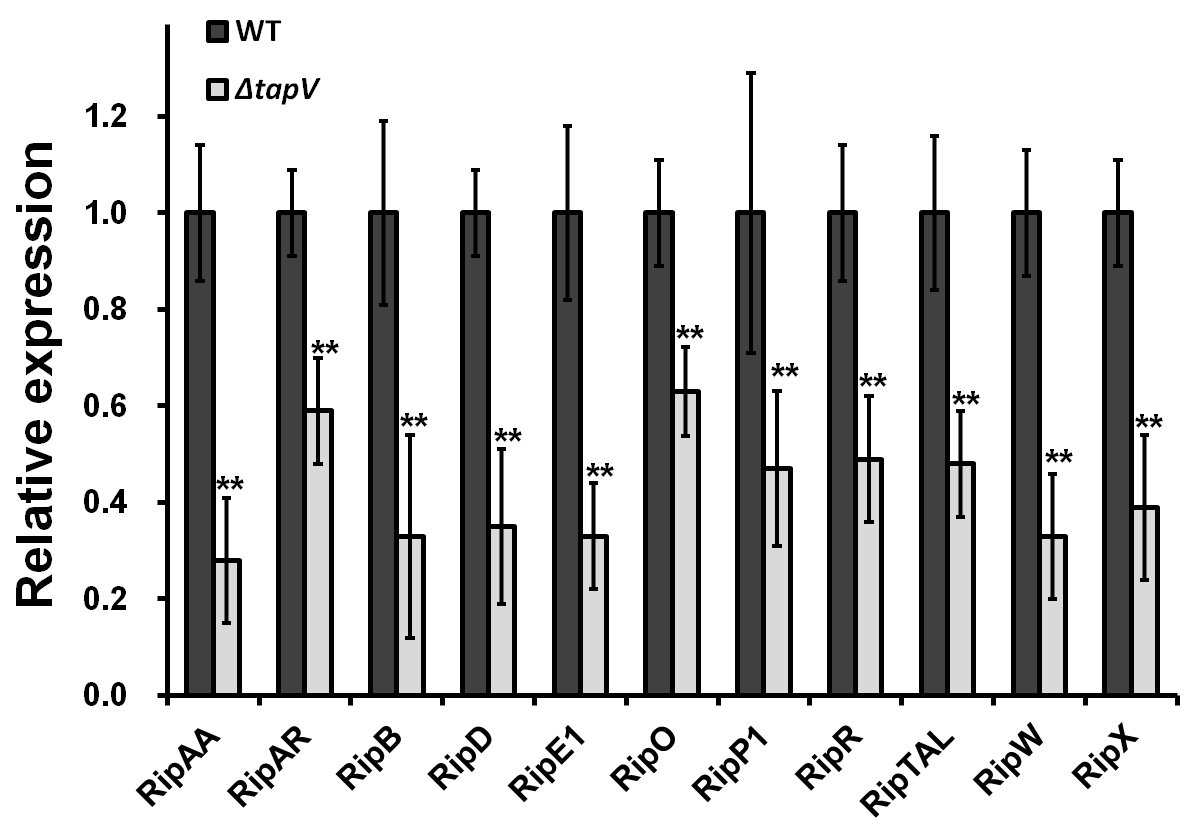


Fig. S1. Relative expression of T3Es genes in *tapV* mutant. Strains were grown in *hrp-*inducing medium to an OD600 as about 0.1 and total RNA was isolated. The cDNA was synthesized using the PrimeScript™ RT Reagent Kit with gDNA Eraser and mRNA levels of representative T3Es genes were determined by qRT-PCR with reference gene as *serC* for normalization. Normalized values of *tapV* mutant were divided with those of wild type strain (WT) and relative values (relative expression) were presented. Mean values of at least three biological replicates were averaged and presented with SD (error bars). Statistical significance between the wild-type strain and *prhP* mutants was assessed using a *post hoc* Dunnett test following ANOVA.. Significance level, ** indicates *P*＜0.01.
