# Supplementary material for: Expression of Ralstonia solanacearum type III secretion system is dependent on a novel type 4 pili (T4P) assembly protein (TapV) but is T4P independent
Source: Mol Plant Pathol. 2020 Mar 20;21(6):777–93. doi: 10.1111/mpp.12930 (PMC7214476; doi:10.1111/mpp.12930)
Supplement: Supplementary file 2 — FIGURE S2 HR test. Approximate 50 µl of bacterial suspension at 108 cfu/ml was infiltrated into tobacco leaves with a blunt‐end syringe. (a) GMI1000, the wild‐type strain, (b) GF0067 (GMI1000, ΔtapV), (c) GF0106 (GMI1000, ΔpilA), (d) GF0108 (GMI1000, Δrsp0189), and (e) distilled water. Development of necrotic lesions was observed periodically and pictures were taken. Each experiment was repeated at least for four times and each treatment contained four plants. The results presented are from a representative experiment, and similar results were obtained in all experiments [file MPP-21-777-s002.docx]

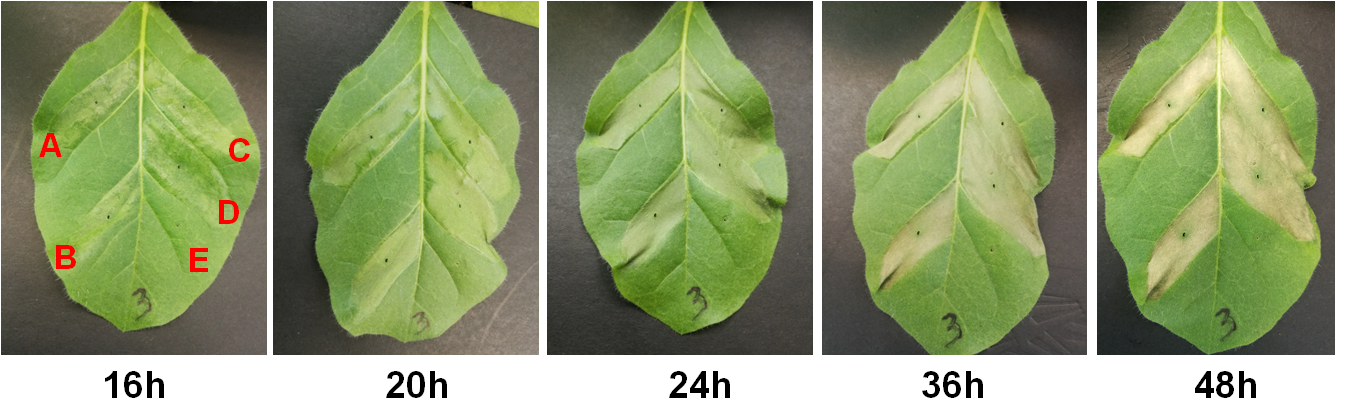


Fig. S2. HR test. Approximate 50 μl of bacterial suspension at 10^8^ cfu ml^-1^ was infiltrated into tobacco leaves with a blunt-end syringe. (A), GMI1000, the wild-type strain; (B), GF0067 (GMI1000, *ΔtapV*); (C), GF0106 (GMI1000, *ΔpilA*); (D), GF0108 (GMI1000, *Δrsp0189*); (E), distilled water. Development of necrotic lesions was observed periodically and pictures were taken. Each experiment were repeated at lest for four times and each treatment contains four plants. The results presented are from a representative experiment, and similar results were obtained in all experiments.
