# Supplementary material for: Expression of Ralstonia solanacearum type III secretion system is dependent on a novel type 4 pili (T4P) assembly protein (TapV) but is T4P independent
Source: Mol Plant Pathol. 2020 Mar 20;21(6):777–93. doi: 10.1111/mpp.12930 (PMC7214476; doi:10.1111/mpp.12930)
Supplement: Supplementary file 3 — TABLE S1 Primers used in this study [file MPP-21-777-s003.docx]

**Table S1. Primers used in this study**

| primer | sequence | Reference source |
| --- | --- | --- |
| tapVA1B | CTggatccAGATCGAGTCCATCATCCGT | This study |
| tapVB1C | GCCGTACGGCGCAGTGCCGACTTCGACTCCGTGATCTT | This study |
| tapVA2C | AAGATCACGGAGTCGAAGTCGGCACTGCGCCGTACGGC | This study |
| tapVB2H | CtaagcttGCCTTCGAGCAGCGGTGCG | This study |
| tapVB3K | GCGGTACCTCGGTCGAATACATCGTC | This study |
| tapVB4K | CTGGTACCGCTCAGATCGATCTCGGCCT | This study |
| pilAA1B | GCGGATCCGGTGCTGCTGGTCTACATC | This study |
| pilAB1C | ACTACAGCAGCTAGCTTAGCGGTTTCTCCCCGAAAGCGTT | This study |
| pilAA2C | AACGCTTTCGGGGAGAAACCGCTAAGCTAGCTGCTGTAGT | This study |
| pilAB2H | CGAAGCTTCCACGTGATTCGGCTGATTC | This study |
| pilAB3H | GCAAGCTTAGCTTACAGCTTAGC | This study |
| rsp0189A1B | GCGGATCCTGGGCCAAGTGCTGATCC | This study |
| rsp0189B1C | GCGCCCGATCGATGGTGTGCGCCTCACCCTTCATTCCCCGT | This study |
| rsp0189A2C | ACGGGGAATGAAGGGTGAGGCGCACACCATCGATCGGGCGC | This study |
| rsp0189B2H | GCAAGCTTCATGGTGATCCGCCAGGC | This study |
| glmsdown | GCGCTCAAGCTCAAGGAGATC | Zhang *et al*., 2011 |
| Tn7R | CACAGCATAACTGGACTGATTTC | Choi *et al*., 2005 |
| lacZR1 | GCGCCATTCGCCATTCAGGCT | Zhang *et al*., 2013 |
